# Life history correlates of fecal bacterial species richness in a wild population of the blue tit *Cyanistes caeruleus*

**DOI:** 10.1002/ece3.1384

**Published:** 2015-01-22

**Authors:** Clare McW H Benskin, Glenn Rhodes, Roger W Pickup, Mark C Mainwaring, Kenneth Wilson, Ian R Hartley

**Affiliations:** 1 Lancaster Environment Centre, Lancaster UniversityLancaster, LA1 4YQ, UK; 2 Centre for Ecology and Hydrology, Lancaster Environment CentreLibrary Avenue, Bailrigg, Lancaster, LA1 4AP, UK; 3 Division of Biomedical and Life Sciences, School of Health and Medicine, Lancaster UniversityLancaster, LA1 4YQ, UK

**Keywords:** 16S rRNA, bacterial species richness, fecal microbial community analysis, life-history traits, operational taxonomic unit, PCR-TGGE

## Abstract

Very little is known about the normal gastrointestinal flora of wild birds, or how it might affect or reflect the host's life-history traits. The aim of this study was to survey the species richness of bacteria in the feces of a wild population of blue tits *Cyanistes caeruleus* and to explore the relationships between bacterial species richness and various life-history traits, such as age, sex, and reproductive success. Using PCR-TGGE, 55 operational taxonomic units (OTUs) were identified in blue tit feces. DNA sequencing revealed that the 16S rRNA gene was amplified from a diverse range of bacteria, including those that shared closest homology with *Bacillus licheniformis, Campylobacter lari*, *Pseudomonas* spp., and *Salmonella* spp. For adults, there was a significant negative relationship between bacterial species richness and the likelihood of being detected alive the following breeding season; bacterial richness was consistent across years but declined through the breeding season; and breeding pairs had significantly more similar bacterial richness than expected by chance alone. Reduced adult survival was correlated with the presence of an OTU most closely resembling *C. lari*; enhanced adult survival was associated with an OTU most similar to *Arthrobacter* spp. For nestlings, there was no significant change in bacterial species richness between the first and second week after hatching, and nestlings sharing the same nest had significantly more similar bacterial richness. Collectively, these results provide compelling evidence that bacterial species richness was associated with several aspects of the life history of their hosts.

## Introduction

Empirical studies documenting the intestinal bacterial flora of wild birds are sparse and have concentrated disproportionately on either those species considered most likely to transmit bacteria to humans or acquire bacteria from human sources, such as gulls (*Larus* spp.) and feral pigeons (*Columba livia*; reviewed by Benskin et al. 2009); or on surveys of dead birds recovered following disease outbreaks, particularly those in visible locations such as urban parks or gardens (Keymer 1958; Faddoul et al. 1966; Kirkwood et al. 1995). Increasingly, it is acknowledged that the vertebrate gastrointestinal microbiome influences, and is influenced by, a wide range of factors, including ecological variables such as diet and habitat (Blanco et al. 2006; Janiga et al. 2007; Literak et al. 2012); micro-environmental factors associated with the anatomical structure of the digestive tract (Berg 1996; Stevens and Hume 1998); and, in birds, by bacteria from the reproductive tract (Lombardo et al. 1999; Stewart and Rambo 2000; Hupton et al. 2003). Shifts in the bacterial community structure have been documented to have important consequences for host health in humans (Kau et al. 2011; Wu et al. 2011) and may have an equally important impact on host fitness in birds. Thus, documenting the normal microflora of the avian gut by identifying bacterial presence and richness, as well as factors affecting their distribution in a natural population of birds, is of fundamental importance if we are to fully understand the complexities of bacterial interactions within birds, and appreciate whether and how communities of gut bacteria influence the host life history.

There are few data concerning the factors that influence the composition and dynamics of the gastrointestinal microbial communities in birds, although there is evidence of similarities of microflora within family groups, and consistency within individuals over time. In nestling tree swallows *Tachycineta bicolor*, for example, the species richness of cloacal microflora was more similar in nestlings reared in the same nest, than between individuals reared in different nests (Lombardo et al. 1996), possibly because of genetic effects on host microbial colonization, or due to nestlings from the same brood sharing the same environment (Mills et al. 1999). A partial cross-fostering experiment involving nestling blue tits *Cyanistes caeruleus* and great tits *Parus major* found a strong environmental effect on the assemblages of cloacal bacteria, as well as significant variation between individuals, suggesting possible genotype-by-environment interactions (Lucas and Heeb 2005). Additionally, a longitudinal study of captive zebra finches *Taeniopygia guttata* demonstrated that individual birds showed consistency in their gut microflora over a period of 10 weeks, as estimated through fecal analysis, although there was significant variation between individuals despite sharing a common aviary environment (Benskin et al. 2010). Currently, the fitness consequences of this variation in gut bacterial species richness are largely unknown.

The aim of this study was to quantify variation in the bacterial species richness found in the feces of a wild population of blue tits using the DNA fingerprinting technique temperature gradient gel electrophoresis (TGGE). These data were used to determine whether species richness was related to several life-history traits including age class (adults vs. nestlings), sex, and various components of reproductive success, as well as several other spatial and temporal variables. In particular, the hypotheses tested were that (1) fecal bacterial richness of an individual within a family is influenced by both genotype and environment, such that families will have a similar richness because they share at least one of these factors; (2) given that that genotype and environment influence fecal microbial richness, and that both were consistent over the period of the study, bacterial species richness of adults will be stable from one year to the next; (3) nestlings will rapidly develop increasing bacterial species richness over the first 2 weeks of their lives, due to accumulation of new bacterial species from the environment; and (4) if the microbial community includes pathogenic bacteria or beneficial microbes that this will be reflected in the host's life-history traits.

## Materials and Methods

### Study site and sample collection

Data were collected from a population of blue tits breeding in nest boxes near Lancaster University, UK (54°0′N, 02°78′W) from April to June of 2007 and 2008. Nest boxes (150 × 150 × 200 mm) were positioned in woodland areas, mounted on trees, in a grid-like pattern at intervals of approximately 50 m, with the majority of the nest boxes being 1.5 to 2 m from the ground, and facing in either a southerly or easterly direction to provide shelter from the prevailing winds, although some local variation existed due to topographical features and habitat structure. Nest boxes were visited 1–3 times per week, beginning on April 1st, to establish standard reproductive variables such as the onset of egg-laying, clutch size, hatch date, hatching success, and fledging success (Leech et al. 2001). Nestlings were ringed with numbered, metal British Trust for Ornithology (BTO) rings 6 days after hatching, while tarsus length and mass were obtained 14 days after hatching. Meanwhile, adults were trapped in the nest while provisioning nestlings and were ringed and measured following protocols described in Mainwaring et al. (2010). Adults were fitted with a unique combination of color rings so that individuals could be identified without the need to recapture them.

Fecal samples were collected from breeding adult blue tit pairs, and from their nestlings when they were aged 7 (±1) and 14 (±1) days after hatching, during the normal handling of the birds as part of the ringing and measuring process. Samples were immediately stored in Eppendorf tubes and frozen at −20°C within 6 h of collection.

### Sample processing

#### DNA extraction and amplification

Total community DNA was isolated from blue tit feces using the QIAamp DNA Stool Mini Kit (Qiagen Ltd, Crawley, UK) incorporating minor modifications similar to those adopted by Wehausen et al. (2004). Briefly, fecal samples were suspended in 1.4 mL of the extraction buffer provided, added to FASTPREP lysing matrix B tubes (MP Biomedicals, UK) and homogenized using a FASTPREP machine (MP Biomedicals, Santa Ana) for 45 s at 6.5 m/s. Samples were centrifuged at 16000 g for two minutes to pellet beads and fecal detritus. The supernatant containing the bacterial DNA was decanted and used subsequently according to the manufacturer's instructions.

Bacterial 16S rRNA genes were partially amplified by nested PCR using primer pair pA/pH' (Edwards et al. 1989), followed by amplification using the universal primers F984/R1378, with the forward primer incorporating a 34 bp GC-clamp (Muyzer et al. 1993). PCR amplifications for each nested PCR were optimized using the Failsafe PCR system (EpiCentre, Cambridge) in 50 *μ*L volumes, such that initial reactions received the following: 25 *μ*L 2× Failsafe premix buffer E, 1 *μ*L (20 pmol) of each primer, 0.5 *μ*L *Taq* polymerase mixture, 12.5 *μ*L sterile molecular biology grade water (DNA-free water; Sigma-Aldrich Company Ltd, Poole, UK), and 10 *μ*L of DNA. Nested reactions differed only in receiving 2× Failsafe premix buffer F, 1 *μ*L DNA and the volume was made up to 50 *μ*L with DNA-free water. Positive controls contained genomic DNA from *E*. *coli* K12 in place of sample DNA, while negative controls contained water instead of sample DNA. All PCR amplifications were carried out in a PX2 thermal cycler (Thermo Electron, Loughborough, UK). Initial denaturation was carried out for 4 min at 94°C, followed by 35 cycles of denaturing at 94°C for 1 min, annealing at 55°C for 1 min, and elongation at 72°C for 1 min. Nested reactions were denatured for 4 min at 94°C, and cycling was performed at 94°C for 1 min, 60°C for 45 s, and 72°C for 2 min for 35 cycles. All amplifications were completed with a final elongation at 72°C for 10 min.

#### Temperature gradient gel electrophoresis (TGGE) and DNA sequencing

TGGE was carried out using the TGGE Maxi system (Whatman, Biometra, Germany). Electrophoresis was performed using 3 *μ*L of each amplification product in polyacrylamide gels (6% polyacrylamide, 7M urea, 10% formamide and 2% glycerol) with 1 × TBE buffer (40 mmol/L tris-borate-EDTA, pH 8.0), using a multistep program. Step one lasted for 10 min at 300 V with a constant temperature of 20°C across the gel, to enable samples to migrate into the gel. The second step consisted of establishing a temperature gradient from 39.5°C to 47.5°C without applying voltage, to allow the temperature gradient to stabilize prior to electrophoresis. Once the temperature gradient had stabilized, electrophoresis was performed for 3 h at 300 V. Gels were subsequently stained with SYBR Gold (Invitrogen, Paisley, UK) for 30 min and visualized on a UV trans-illuminator (320 nm).

For DNA sequencing, each band of interest was excised from gels under UV light, using a sterile scalpel blade, and placed into individual sterile Costar Spin-X centrifuge tube filters (Corning Inc., Tewksbury, USA) containing 40 *μ*L elution buffer (QIAamp DNA Stool Mini Kit). DNA was then extracted by centrifugation at 13,000 rpm for 10 min, and SYBR gold stain was by ethanol precipitation. The purified DNA (20 *μ*L) was amplified with primers F984/R1378, using the PCR conditions described above.

PCR products were purified with the QIAquick PCR purification kit (Qiagen Ltd, Crawley, UK), following which, 3 *μ*L of each product was cloned into the pDrive cloning vector using the QIAGEN A-addition Kit and the QIAGEN PCR Cloning Kit. Plasmid DNA was extracted from bacterial clones using the QIAprep Spin Miniprep Kit, according to the manufacturer's instructions. The presence of cloned inserts was confirmed by restriction digestion using the enzyme *Eco*RI (Abgene) followed by electrophoresis for 1 h at ∽100 V in 0.7% w/v agarose gels. The DNA sequence from these 16S clones was determined by Qiagen Genomic Services (Germany) by single read. Sequences were compared with those in the GenBank database with the BLASTn search program (Altschul et al. 1990; http://blast.ncbi.nlm.nih.gov/Blast.cgi). Sequences described have been deposited into GenBank and assigned Accession Numbers AB700978–AB701013 (TCbt1-36), AB734778 and AB734779 (TCbt37-38).

### Estimation of microbial species richness

The positions of bands on TGGE gels were normalized using a control sample as an internal marker, and the molecular weight ladder Lambda *Eco*RI/*Hind*III (Advanced Biotechnologies, Surrey) as an external reference standard, to permit comparisons between gels. Bacterial species richness in each sample was measured by counting the number of bands found in each lane, with the assumption that each band represents a different 16S rRNA gene sequence (and consequently a different bacterial species), and therefore different operational taxonomic units (OTUs; Atlas and Bartha 1986). A binary matrix was produced for each sample by noting the presence/absence of each OTU.

### Statistical analysis

The data were analyzed using generalized linear models (GLMs) with quasipoisson errors and a log link function, or generalized linear mixed models with Poisson errors with nest-box identity (*Family*, see below) as a random term (McCullagh and Nelder 1989). Data analysis was performed using the *S-Plus 8.0* (Insightful Corp., Washington) and R (v. 3.0.1; R Core Team 2013) statistical packages. The analyses were designed to determine the factors associated with variation in bacterial species richness (*Richness*; number of OTUs per fecal sample) within and between individuals. Potential explanatory variables were *Year* (2007 or 2008), *Family* (a nest-box/family identifier), *Wood* (three adjacent habitat blocks), *Bird Age–Sex* class (four levels: male and female adults, and 7- and 14-day-old chicks), *Date Sampled* (days since the start of the breeding season, April 1st), *Time* (hour of sampling), and *Bird Identity* (as determined by BTO ring numbers).

Several other variables were available for either the adults or the nestlings, which were included in separate age-specific analyses. For the adults, these were *Sex* (male or female), *Mass* (body mass in grams), *Wing Length* (mm), *1st Egg Date* (date the clutch was initiated), *Clutch Size* (number of eggs in the clutch), *percentage of Nestlings Fledged* (the proportion of hatched eggs that produced a successful fledgling), and *Survival* (whether or not the bird was detected alive in the following breeding season). *Survival* was estimated by surveying the breeding population for color-ringed adult birds, and capture at the nest in some cases, in the year following the assessment of fecal bacteria. If birds were detected alive in 2008 or 2009, following the breeding seasons in 2007 and 2008, respectively, they were categorized as having survived. If they were not detected, they were assumed to have died. Adult blue tits generally breed in the same areas in consecutive years, so while we may have incorrectly designated some surviving birds as nonsurvivors, the number of these is likely to be small. We used our estimate of adult survival as the dependent variable in GLMs (binomial errors and a logit link function, tested against the chi-square distribution) to explore the factors relating to survival between breeding seasons. For nestlings, additional variables were *Nestling Age* (7 or 14 days old), *Mass* (at day 14 after hatching) and *Survival to Fledging* (fledged successfully from the nest or not). Separate analyses were conducted for nestlings and adults because the two age-classes were associated with different explanatory terms. In all cases, maximal models were first considered, and then nonsignificant terms were removed from the model one at a time, using a stepwise-deletion process, until only significant terms remained in the model (Crawley 2002). Parameter estimates are for when significant terms are included last in the model.

## Results

### Bacterial species richness within the blue tit population

**Figure 1:**
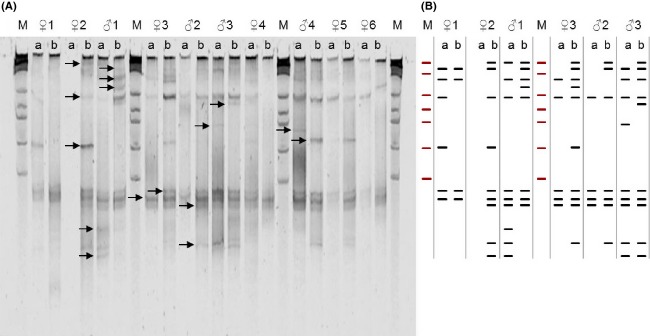
Temperature gradient gel electrophoresis (TGGE) image of bacterial assemblages in faecal samples from ten blue tits, showing variation in paired samples from six female (♀1–6) and four male blue tits (♂1–4), from the 2007 and 2008 breeding seasons (A and B respectively). Arrows indicate the relative positions of the bands detected. M denotes lanes containing the marker *λ*HindIIIEcoR1. A schematic of banding patterns is shown to the right of the gel image.

Fecal samples were collected from a total of 27 families; 17 in 2007 (55 nestlings and 34 adults); and 10 in 2008 (33 nestlings and 20 adults; samples were often not obtained from entire broods). Amplicons from 230 fecal samples (one from each adult, two from each nestling at 7- and 14-day old) were separated by TGGE, which resulted in a total of 55 different bands (OTUs). Thirty-eight of these bands were successfully excised, re-amplified, cloned and sequenced, resulting in partial 16S rRNA gene sequences, ranging between 376 and 439 bp in length. Five of the bands were excised in duplicate (two bands with the same alignment on two different gels) to test for reproducibility of fragment identification between gels. The sequences of each of the five duplicates were in agreement (see supplementary data). This resulted in 35 sequences with ≥97% similarity with a known sequence in the GenBank database and three sequences with ≤96% similarity. The identity of closest homologous sequences, their annual presence and the frequency of each identified OTU is presented in Table1, while an example gel is shown in Figure1.

**Table 1:** Bacterial species detected in the faeces of blue tits in Lancaster, UK, and the frequency with which they occurred. Homologues were identified by analysis of partial 16S rRNA gene sequences using the BLASTn program

TCbt clone no.	Sequence length obtained (bp)	Presence (+) or absence (−) by year		Frequency in present population (%; *n* = 142)	Closest 16S rRNA gene homologue(s) in Genbank [accession no.] (% nucleotide identity)	Origin of homologous sequences (reference)
		2007	2008			
TCbt1	434	+	−	30.3	TCbt7 & TCbt18 (100%)	Present study
TCbt2	413	+	+	40.1	*Campylobacter lari* spp. [e.g. HM007571] (99%)	Various human gastroenteritis patients (UDS)
TCbt3	+	+	−	2.1	No homology >95%	–
TCbt4	419	+	+	49.3	No homology >96%	–
TCbt5	437	+	+	50.0	No homology >96%	–
TCbt6	410	+	+	8.5	*Lactobacillus sp*. [NR104979.1] and uncultured bacterium [HM335530] (99%)	Lactobacillus aviarius subsp. araffinosus (UDS); Skin microbiome (Kong et al. 2012)
TCbt7	417	+	+	14.1	TCbt1 & TCbt18 (100%)	Present study
TCbt8	412	−	+	0.7	*Serratia* spp. [KJ095676] & *Rahnella* spp. [JN656281] (100%)	Unknown source (2014 UDS); Ips sexdentatus; Coleoptera (Sevim et al. 2012)
TCbt9	411	−	+	4.2	Uncultured bacterium [JF168221] (100%) & TCbt14 (99%)	Skin microbiome (Kong et al. 2012); present study
TCbt10	411	−	+	1.4	Uncultured bacterium clone [EU777815] (100%)	Polar bear faeces (Ley et al. 2008)
TCbt11	412	−	+	19.0	*Rickettsiella* spp. [HQ640943] (99%)	*Agroites* sp. wireworm pathogen (Leclerque et al. 2011)
TCbt12	436	+	−	1.4	*Pseudomonas* spp. [KM058081.1] (99%)	Unknown source (UDS)
TCbt13	407	+	−	4.9	Uncultured rumen bacterium clone [GU304476.1] (98%); *Atopobium parvulum* [KC999391] (97%)	Uncultured rumen bacterium (UDS); Atopobium parvulum (UDS)
TCbt14	411	−	+	0.7	TCbt9 and uncultured bacterium [JF168221] (99%)	Present study; skin microbiome (Kong et al. 2012)
TCbt15	411	+	+	72.5	*Bacillus licheniformis* strains [KJ495982; KJ126941; KJ126938] (99%)	Unknown source (2014 UDS)
TCbt16	434	−	+	15.5	Uncultured bacterium clone [HM341167] (99%)	Skin microbiome (Kong et al. 2012)
TCbt17	419	+	−	37.3	*Staphylococcus* spp. [KC213932] (99%)	Freshwater roach (2013 UDS)
TCbt18	383	+	+	29.6	TCbt1 & TCbt7 (100%)	Present study
TCbt19	412	−	+	0.7	Uncultured *Moraxella* spp. [JN792320] (99%)	Finless porpoise faeces (McLaughlin et al. 2012)
TCbt20	412	+	−	2.8	*Pseudomonas* spp. [DQ904607] (99%)	Wireworm gut (2006 UDS)
TCbt21	409	+	+	11.3	Uncultured bacterium clone [JQ451662] (99%)	Environmental samples (2012 UDS)
TCbt22	439	−	+	1.4	Uncultured bacteria [EU540256; DQ264573] (99%)	Human skin (Grice et al. 2008); subsurface groundwater (DeSantis et al. 2007)
TCbt23	410	−	+	1.4	Uncultured Lactobacillus sp. (99%)	Silkworm midgut (Yuan et al. 2005, UDS) fasted chicks (UDS)
TCbt24	412	−	+	2.8	*Erwinia persicina*. [AB934973] (100%)	Beer brewing process (2014 UDS)
TCbt25	376	+	−	2.8	*Pseudomonas veronii* [KJ726603] (100%)	2014 UDS
TCbt26	412	+	−	2.8	*Pseudomonas* spp. [KF263621] (97%)	Antarctic soil bacteria (Goh and Tan 2012)
TCbt27	414	+	+	4.2	*Campylobacter lari* [KF703982] (99%)	American crows (Weis et al. 2014)
TCbt28	436	+	+	4.9	Uncultured *Pseudomonas* [DQ144425] (100%)	Soil (Hjort et al. 2007)
TCbt29	411	+	−	3.5	*Salmonella* enterica subsp. enterica [CP003836] (99%)	Unknown source (2014 UDS)
TCbt30	435	+	+	13.4	Uncultured and cultured *Erwinia* spp. [JN793861; CU468135] (99%)	Environmental sources (UDS and Kube et al. 2008)
TCbt31	412	−	+	4.2	Uncultured bacteria [HM557599] [JX559723] (99%)	Leaf cutting ants (Suen et al. 2010); green tea (UDS)
TCbt32	418	−	+	2.8	*Salmonella* spp. [CP003416] (98%)	Faeces (Le Bars et al. 2012)
TCbt33	403	+	+	4.2	Uncultured bacterium [EU136788] (98%)	Environmental samples (Jones et al. 2008)
TCbt34	436	+	+	4.2	Uncultured [JF829474] and *Microlunatus* spp. [NR_116819] (98%)	Soil (Kampfer et al. 2010; Talia et al. 2012)
TCbt35	381	+	+	28.2	Uncultured bacterium [FM872481] (99%)	House floor dust (Taubel et al. 2009)
TCbt36	437	+	+	4.2	Uncultured bacterium [JF729045] (99%)	2011 UDS
TCbt37	433	+	−	1.4	Uncultured *Microbacterium* spp. [KC922070] (99%)	Rabbit (2014 UDS)
TCbt38	433	+	−	2.1	*Arthrobacter* spp. [KJ870017] (99%)	Picrorhiza rhizosphere (2014 UDS)

UDS = Unpublished direct submission of DNA sequence.

**Figure 2:**
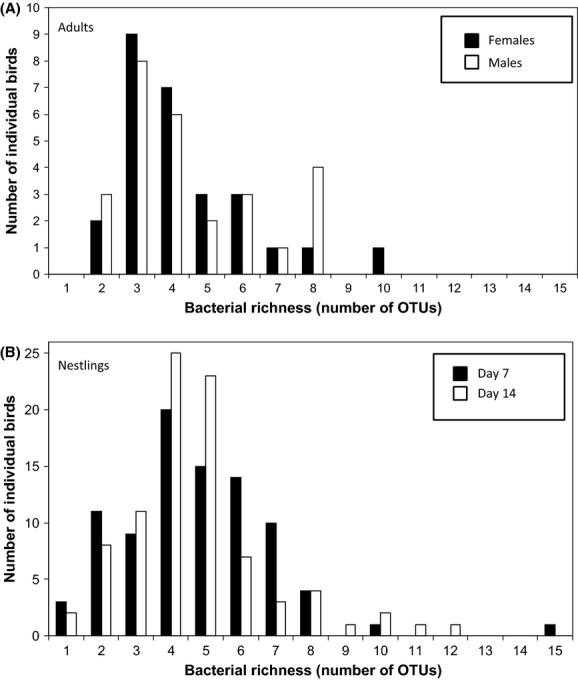
Frequency distribution of bacterial species richness in blue tits, as determined by the bacterial species richness recorded within individual adults (A) and nestlings (B). There was no significant difference in bacterial species richness between adult male and female blue tits (*F*_1,52_ = 0.02, *P *=* *0.888) nor between nestlings aged 7 or 14 days after hatching (*F*_1,174_ = 0.03, *P *=* *0.859).

Overall, bacterial species richness (as estimated by the number of bacterial OTUs in a bird's feces) did not differ significantly between the four age–sex classes of birds (GLM: *Age–Sex* class: *F*_3,226_ = 0.337, *P *=* *0.80; mean *Richness* ± SD: adult males = 4.37 ± 1.86; adult females = 4.44 ± 1.97; 7-day-old chicks = 4.75 ± 2.18; 14-day-old chicks = 4.69 ± 2.06; Fig.2). However, bacterial richness did differ significantly across families and there were significant interactions between family identity and both the year sampled (2007 or 2008) and bird age–sex class (GLM: *Family*: *F*_23,202_ = 4.90, *P *<* *0.0001; *Year Sampled*: *F*_1,202_ = 0.00, *P *=* *1.00; *Age–Sex*: *F*_3,202_ = 0.95, *P *=* *0.42; *Family* × *Year Sampled*: *F*_2,131_ = 21.33, *P *<* *0.0001; *Family *× *Bird Age–Sex*: *F*_69,131_ = 1.44, *P *=* *0.038). Thus, there were significant differences between families in their bacterial richness, and the response of families (nest boxes) differed across years and between bird age–sex classes. To explore these effects further, and to take account of age-specific covariates, we next analyzed the data separately for adults and chicks.

### Temporal consistency of bacterial species richness in adults

**Figure 3:**
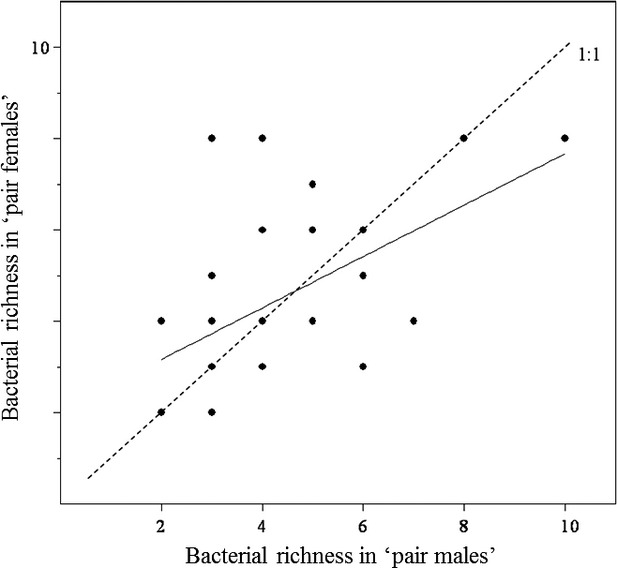
Bacterial species richness in the faeces of blue tit adults in breeding pairs. The species richness in males and females within pairs was significantly, positively correlated (*r* = 0.532, df = 25, *P* < 0.005). The dashed line represents the 1:1 relationship, where partners in each pair would have the same species richness. The solid line represents the linear regression line of best fit. Some dots represent more than one pair.

**Figure 4:**
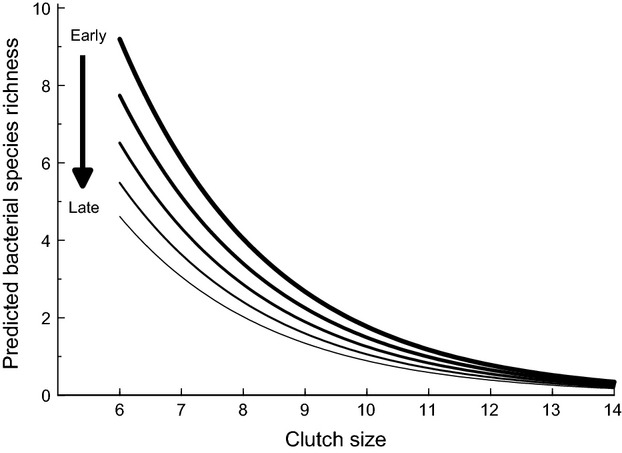
Relationship between bacterial species richness and both the number of chicks reared (*Clutch Size*) and the date that the clutch is initiated (*1st Egg Date*). Lines represent the predicted values from the model for clutches initiated at various points in the season (*1st Egg Date*: thickest line = April 24th; thinnest line = May 2nd).

Analysis of data pertaining to the adult blue tits showed a highly significant effect of *Family* on fecal bacterial species richness (*Richness*), indicating that breeding pairs varied in the number of bacterial OTUs they harbored (Table2). This is reflected in the strong positive correlation between the species richness of matched fecal samples collected from males and females of breeding pairs (Pearson's correlation test: *r *=* *0.532, df = 25, *P *=* *0.0043; Fig.3). In addition, there was a weak, but statistically significant, decline in bacterial species richness with *Date Sampled* (although if two low-richness samples collected very late in June are excluded, this relationship becomes nonsignificant). After accounting for these effects, there were highly significant, and independent, declines in bacterial richness associated with both *1st Egg Date* and *Clutch Size* (Table2; Fig.4). Thus, those birds that bred later in the season or produced larger clutches tended to have lower bacterial richness. There was no significant variation in bacterial species richness of adults associated with any other explanatory terms tested (*Year*, *Wood*, *percentage of Nestlings Fledged*, *Hour Sampled*, *Mass* or *Wing Length*), including *Sex* (Fig.2A).

**Table 2:** Analysis of deviance table for the minimal model of a GLM with quasipoisson errors (log link function), with *Richness* in adult blue tits as the dependent variable

Term	Degrees of freedom	Relationship	Deviance	*F*	*P*
Date Sampled	1	Negative	1.25	4.24	0.0497
1st egg date	1	Negative	5.00	16.92	0.0003
Clutch size	1	Negative	4.86	16.44	0.0004
Family	23		29.68	4.37	0.0002

The main effects in the maximal model were *Year, Wood, Family, Sex, Date Sampled, Hour Sampled, Mass, Wing Length, 1st Egg Date, Clutch Size,* and *percentage of Nestlings Fledged*. Null deviance = 40.35, Null df = 52, Residual deviance = 7.77, Residual df = 26.

Most adult birds were not sampled in both breeding seasons due to them either evading recapture, or because they did not survive until the following breeding season. However, fecal samples and species richness estimates were obtained in both years for four male and five female adults, enabling the repeatability of bacterial species richness across years to be tested. This revealed that species richness was not repeatable across years (*Bird Identity*: *F*_8,9_ = 1.24, *P *=* *0.37).

### Adult survival and bacterial species richness

**Figure 5:**
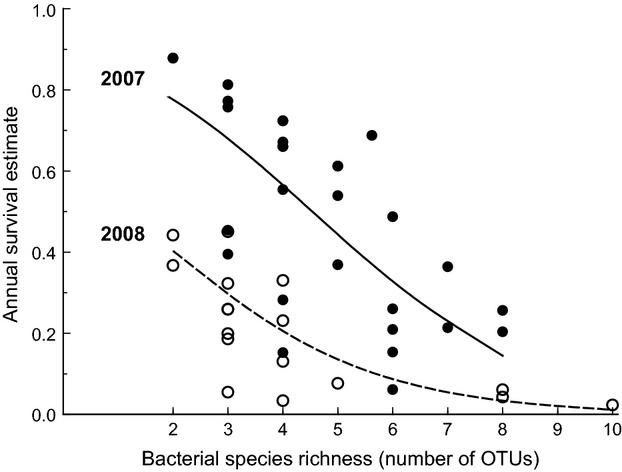
Annual survival of adult blue tits in relation to bacterial species richness and year of sampling showing that survival differed across years and declined with increasing bacterial species richness. Data points and lines are the fitted values from the logistic regression model. Solid symbols and line are for birds sampled in 2007, open symbols and dashed line in 2008.

Annual survival was estimated by quantifying whether the bird was seen in the study area during the year following the sampling period (a binomial value, 0 or 1). Logistic regression indicated that survival was higher for adults sampled in 2007 (53%) than in 2008 (20%) and was positively associated with the percentage of nestlings successfully fledged (Table3). After controlling for these effects, there was a strong negative association between adult survival and fecal bacterial species richness, such that in 2007 predicted annual survival declined from around 80% in birds with the least number of OTUs (two) to <20% in those with the highest (eight OTUs; Fig.5, Table3). There was no association between annual survival and spatial location (*Family* and *Wood*), other nest characteristics (*Date Sampled*, *Hour Sampled*, *1st Egg Date*) or bird life-history traits (*Sex*, *Mass* and *Wing Length*).

**Table 3:** Analysis of deviance table for the minimal model of a GLM with binomial errors (log link function), with inter-annual survival of adult blue tits as the dependent variable

Factor	Degrees of freedom	Relationship	*χ* ^2^	*P*
Year	1	2007 > 2008	5.57	0.0183
% Nestlings Fledged	1	Positive	4.88	0.0272
Richness	1	Negative	7.08	0.0078

The main effects in the maximal model were *Year*, *Wood*, *Richness*, *Sex*, *Date Sampled*, *Hour Sampled*, *Mass*, *Wing Length*, *1st Egg Date*, *Clutch Size,* and *percentage of Nestlings Fledged*. Null deviance = 73.00, Null df = 53, Residual deviance = 55.02, Residual df = 50.

To test for band-specific patterns in relation to survival, a logistic regression model was analyzed in which annual survival was the dependent variable and the explanatory terms were the binomial codings for the presence (1) or absence (0) of specific TGGE bands. Because there is a 5% probability of producing a significant result by chance alone, if all 34 TGGE bands present in the adult population were tested, two significant results would be expected due to chance. Therefore, to reduce the probability of type I errors, only those nine bands that occurred in >20% of the adult population were tested (*Bands 3, 4, 6, 14, 18, 19, 21, 50,* and *51*), which at a 5% error rate would result in <1 false positive on average. Of these nine bands, *Band 3* (TCbt2, *Campylobacter lari* – prevalence = 28%) and *Band 51* (TCbt38, *Arthrobacter* spp. – prevalence = 41%) were significantly correlated with survival probability (TCbt2: parameter estimate, *b *± SE = −2.219 ± 0.884; 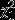 = 8.07, *P = *0.0045; TCbt38: *b * ± SE = 1.807 ± 0.743; 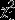 = 7.06, *P *=* *0.0079). Annual survival was reduced in birds harboring TCbt2, but enhanced in those with TCbt38, and these effects were additive (interaction term: 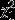 = 0.69, *P *=* *0.41), such that birds harboring TCbt2, but not TCbt38 suffered 100% annual mortality, whereas those hosting TCbt38, but not TCbt2 suffered just 25% annual mortality (Table4). TCbt2 prevalence increased with the bacterial species richness (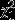 = 6.74, *P *=* *0.0094), but TCbt38 prevalence did not (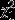 = 0.19, *P *=* *0.66); thus, the former relationship may help to explain why annual survival declined with increasing bacterial richness (and hence increased prevalence of deleterious *C. lari*). Consistent with this, when bacterial richness was included in the survival model, TCbt2 did indeed become nonsignificant, whereas TCbt38 retained its significance (*Richness*: 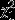 = 6.92, *P *=* *0.0085; TCbt38: 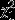 = 4.52, *P *=* *0.034; TCbt2: 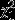 = 1.56, *P *=* *0.21).

**Table 4:** Mean survival rates (%) of adult blue tits (*n* = 54) carrying TGGE OTUs TCbt2 (*Campylobacter lari*) and TCbt38 (*Arthrobacter* spp)

		TCbt2		
		Present	Absent	Total
TCbt38	Present	37% (27)	75% (12)	55% (39)
	Absent	0% (5)	30% (10)	31% (15)
	Total	20% (32)	49% (22)	41% (54)

### Bacterial species richness in nestlings

The number of OTUs detected in fecal samples from nestlings varied significantly between families (GLM, *Family*: *F*_23,152_ = 7.87, *P *<* *0.0001). GLMMs were therefore used to account for this potential pseudo-replication across families by including *Family* as a random term in the model. After doing so, no effect of chick age (7 or 14 days old) on bacterial species richness (*Nestling Age*: *F*_1,87_ = 0.077, *P *=* *0.78; Fig.2B) was found, which is inconsistent with the hypothesis that nestlings will rapidly develop increasing bacterial species richness over the first 2 weeks of their lives. Moreover, bacterial species richness also did not vary with *Year*, *Wood*, *Age*, *Date Sampled*, *Mass*, *1st Egg Date*, *Clutch Size,* and *percentage of Nestlings Fledged*. Nestling survival to fledging was uniformly high at 96% (*n* = 176 chicks) and “survival” to 1-year old was uniformly low at 1%, presumably in part due to postfledging dispersal. As a consequence, survival analysis would not be meaningful and was not conducted.

## Discussion

In making assessments from PCR-TGGE-derived data, it is pertinent to consider possible limitations associated with this technique. PCR-based fingerprinting techniques are subject to the drawbacks and limitations of PCR itself. The most significant of these include that amplification can be inhibited by contaminants that co-extract with DNA; that there can be preferential or selective amplification of DNA from mixed communities; and there can be formation of chimeric or heteroduplex DNA molecules (Nannipieri et al. 2003). Other more TGGE-specific limitations are also important. In the present study, we have interpreted each separate band as an OTU when in fact separate OTUs are capable of comigrating and being indistinguishable (Ercolini, 2004). It is important therefore to stress that our interpretations are subject to these limitations. Despite these limitations, TGGE remains a very useful tool for microbial richness assessments and equally important to note is that even the very latest next generation technologies are also subject to PCR bias (Schwartz et al. 2011; Lee et al. 2012). Thus, we are confident that our methods allow us to objectively examine the fitness consequences of bacterial species richness.

Bacterial species richness in the feces of wild adult and nestling blue tits, collected during the breeding season, were analyzed using 16S rRNA gene sequence variability (for a comparison with traditional culture methods, see Benskin et al. 2010). The species richness of bacteria detected within the feces was used as a proxy for bacterial species richness within the GI tract (Mead 1997; Marchesi 2010), to test whether this was related to a variety of temporal, familial and age-related factors, as well as individual morphology and survival. In adult blue tits, there was a significant correlation between the fecal bacterial species richness of a breeding pair. Species richness was also negatively related to adult survival until the following breeding season and did not change between years in the small number of adult birds assayed in both breeding seasons. For nestlings, however, there was no significant change in bacterial species richness between the first and second week after hatching. Indeed, nestlings had similar numbers of bacterial OTUs as adults, suggesting that there was no ontogenetic change in bacterial species richness from day 7 onwards, although community composition may have changed. The species richness of fecal bacteria was lower for adults sampled later in the breeding season, whereas for nestlings there was no temporal change.

### Bacterial species richness in blue tit feces

Twenty-six and 27 bacterial OTUs were detected in the population in the 2007 and 2008 breeding seasons, respectively, with fifteen of these being common to both years. Although 55 OTUs were detected across the breeding population, no more than fifteen were detected in an individual. A large proportion of the 55 OTUs were relatively rare, such that >40% of the OTUs occurred in four or fewer individuals. This pattern of high bacterial species richness coupled with a low prevalence of each species has also been found in other passerines, including the cloacae of female towhees *Pipilo maculatus* (57 bacterial species; Klomp et al. 2008), nestling blue tits, and great tits (78 bacterial species; Lucas and Heeb 2005), as well as in the ejaculates of male, and the cloacae of male and female, red-winged blackbirds *Agelaius phoeniceus* (53 bacterial species; Hupton et al. 2003).

Taxonomic assignation of sequenced 16S rRNA gene amplicons was carried out for 38 (69%) of the 55 OTUs (Table1), and sequences were found to share homology with those of both potentially beneficial and pathogenic bacteria. Our interpretation of similarity scores and alignments remains tentative and cautionary as no sequences of over 500 bp were obtained. Consequently, we did not assign potential taxonomies beyond genus level, or at all, when similarity scores were <97%. Furthermore, in numerous cases, several identical scores were obtained for BLASTn alignments and consequently were too numerous to list in Table1. The resulting taxonomic placement of the majority of clone sequences in Table1 is therefore representative of a list of several related or unrelated bacteria. Despite this, the putative identity of a few clones warrants further attention due to their potential importance. The most prevalent OTU (TCbt15) was identified almost unequivocally as being homologous to that of *Bacillus licheniformis* in returned alignments. *B. licheniformis* is a recognized feather-degrading bacterium (Goldstein et al. 2004; Møller et al. 2009; Saag et al. 2011) and was detected in the feces of 72.5% of birds sampled. This is of potential importance if the identification is correct, as plumage bacteria, including *B. licheniformis*, have been shown to influence feather and body condition, as well as coloration (Burtt and Ichida 1999; Gunderson 2008; Gunderson et al. 2009), both of which influence mate choice (Bakker and Pomiankowski 1995; Burley and Fostera 2006). These bacteria may have been introduced to the gut during preening.

Five different clones (TCbt12, 20, 25, 26, and 28) were identified as being 97–100% similar to *Pseudomonas* spp. sequences. *Pseudomonads* have recently been described as the most prevalent of bacteria found in the nests of blue and great tits (Goodenough and Stallwood 2010), and while some *Pseudomonas* species are pathogenic (Levesque et al. 2000; Walker et al. 2002; White et al. 2003), they have also been isolated from the plumage of apparently healthy individuals (Shawkey et al. 2005). A study of pied flycatchers that found *Pseudomonas* to be the most common genus of culturable bacteria, present both on eggshells and in the cloacae of breeding females (Ruiz-de-Castañeda et al. 2011), reported their presence to have no effect on hatching success.

Clones TCbt2 and TCbt27 independently shared 99% homology with *Campylobacter lari*, and collectively suggested that *C. lari* might be present in 45% of the blue tit fecal samples examined. *C. lari* has previously been isolated from gulls (Benjamin et al. 1983; Lu et al. 2008; reviewed in Benskin et al. 2009) and *Campylobacter* spp. can cause diarrhea and vomiting in humans. *C. jejuni* is commonly found in birds and is the most common cause of bacterial gastroenteritis in humans worldwide (Allos 2001). As expected for a potentially pathogenic bacterium, the presence of *C. lari* (TCbt2) in the feces of adult blue tits was associated with a decline in annual survival of more than 50%, especially when TCbt38 (*Arthrobacter* spp.) was absent (Table4). Moreover, there was a positive association between bacterial richness and TCbt2, and when overall bacterial richness was accounted for, TCbt2 no longer explained variation in survival, suggesting that part of the association between bacterial richness and survival was explained by the presence of *C. lari*. Although *Arthrobacter* spp. are commonly found in soils, and some species reportedly have bioremediation properties, they have also been reported in the feces of some wild birds. However, the potential health benefits of *Arthrobacter* spp. have not previously been reported and this merits further investigation.

*Salmonella* species can cause significant disease in wild birds, especially in the winter months when birds gather at garden bird feeders (Kirkwood 1998; Pennycott et al. 2002; Tizard 2004; Benskin et al. 2009). Clones TCbt29 and TCbt32 had closest sequence homology (99%) with *Salmonella* spp. and were present in the population at a combined frequency of just 6.3%. The clone sequence TCbt29 was observed only in the feces of five nestlings from one nest in 2007, while TCbt32 occurred in four nestlings from one nest sampled in 2008. It is noteworthy that of the nine blue tit nestlings harboring bacteria with sequences similar to *Salmonella*, all but one fledged successfully. This finding may have implications for the maintenance of this bacterium in the wider population.

The sequences of clones TCbt1, 7, and 18 aligned most strongly with each other (100%) and did not align with others in the database with a score higher than 96%. Clones TCbt9 and TCbt14 were also 99% related, although they shared 100% homology with a sequence from an uncultured bacterium from the human skin microbiome (Kong et al. 2012). This apparent homology does not invalidate the assumption that each separate band represents a different OTU, due to the disparate sequence lengths obtained and that they were only classified to genus level. However, the fact that these sequences are not closely related to others in the database is evidence of their originating from a previously undescribed environment.

We note that several of the clone sequences align with bacterial homologues associated with the skin microbiome. It is possible that bacterial transfer from handler to bird plumage may have occurred when birds were handled prior to sample collection, and although it is unlikely that contamination would affect feces, this possibility cannot be ruled out. When developing, nestlings slough off a large amount of skin cells that build up in the nest, and it is possible that the blue tits ingested bacteria associated with avian skin and that this is what was detected in the feces.

### Bacterial species richness and host attributes

As birds mature, their bacterial microflora stabilizes as their immune system develops (Knarreborg et al., 2002; van der Wielen et al. 2002); thus, adults might have been expected to have lower bacterial abundance than nestlings. However, bacterial species richness, as measured by the number of OTUs detected in each sample, was not significantly different between adult and nestling blue tits. While in the nest, inoculation of nestlings is limited to their direct environment, food sources, and interaction with parents. Once fledged, exposure to a more extensive and varied environment, and thus a broader spectrum of microbial sources, could result in bacterial richness increasing. However, there was no statistical support for the prediction that bacterial species richness in nestlings would increase with age (van der Wielen et al. 2002; Lu et al. 2003; Gong et al. 2008), as might be expected as inoculation of nestlings increases over time through their exposure to microbes transferred by parents, in food and from the nest environment (Berger et al. 2003; Lucas and Heeb 2005). In contrast, variation in nestling bacterial species richness was evident between families, which may be a reflection of the bacterial abundance being sensitive to diet and other environmental factors, such as bacteria growing in the nest itself (e.g., on feces deposited by the nestlings, on sloughed skin cells and feathers).

The bacterial species richness was lower in adults that laid larger clutches and those that bred later in the season. This could be indicative of a link between bacterial species richness and fitness indices. Producing large clutches is costly and can result in a number of trade-offs between reproductive effort and susceptibility to parasitism, including bacterial parasites (Gustafsson et al. 1997). Thus, birds displaying lower richness may be suffering from a less stable bacterial community (Davis et al. 2007), and hence more susceptible to pathogenic bacteria (Khuel et al. 2005). Although parental quality may affect both timing of reproduction, reproductive investment, and reproductive success (Price et al. 1988), bacterial species richness cannot be treated in isolation as an indication of parental quality in regard to reproductive investment and timing, as the community composition of the species present, rather than species richness, may have an effect on host fitness (Benskin C.McW.H., Rhodes G., Pickup R.W., Mainwaring M.C., Wilson K. & Hartley I.R., unpubl. data).

Wild birds and mammals with relatively high body mass are widely considered to be of higher quality (Kitaysky et al. 1999; Schulte-Hostedde et al. 2004), and in the only analysis performed to date, Klomp et al. (2008) found that body mass of female spotted towhees was positively correlated with cloacal microbial richness. This supports the notion that bacterial species richness may be indicative of host fitness, in terms of higher richness in the gut indicating a more stable bacterial community (Davis et al. 2007). Our data from blue tits offered mixed support for a link between bacterial species richness and host quality, as annual survival of adults declined markedly with increasing species richness, while there was no evidence that nestling survival to fledging was related to species richness, due to uniformly high fledging rates. Further experimental investigations would be required to quantify the effect of bacterial species richness on the different components of host fitness, such as whether varying body condition is a cause or a result of bacterial species richness, whether individuals in poor condition are more likely to succumb to bacterial infection, and how bacterial species richness affects fitness relative to host age.

### Sources of variation in fecal bacterial species richness

We found significant variation in fecal bacterial species richness between family groups, which could be attributed to both environmental and host-genetic factors. Individual family groups would be exposed to both different local environments and family host genetics. Nestlings predominantly acquire microbes from food supplied by the parents, but also from ingestion of nest material and through coprophagy (Kyle and Kyle 1993; Mills et al. 1999; Berger et al. 2003). In species with altricial young, parental provisioning of food provides a route of horizontal transmission of microbes through ingestion of adult saliva (Kyle and Kyle 1993). Nestlings within the same nest box were in constant close proximity to one another and would thus be exposed to the same microorganisms derived from both parents and siblings, and those which naturally colonized the nest from the air (Mills et al. 1999; Lucas and Heeb 2005). Genetic similarities between siblings may have contributed to their development of similar internal environments that favoured the growth of similar types of microbes (Mills et al. 1999), which could explain the differences detected in the structure of bacterial assemblages between broods. Similar nest-specific patterns of microbial assemblages have been detected in nestling tree swallows, where a discriminant analysis correctly paired nineteen of 22 nestlings with their brood of origin based on the community structure of their cloacal microflora (Lombardo et al. 1996). Cross-fostered nestlings of two tit species (blue and great) reared in the same nest could not be distinguished by differences in their microbial assemblages, whereas the structure of bacterial communities in nonmanipulated nestlings was shown to be significantly different between the two tit species (Lucas and Heeb 2005). This suggests an association between the nest environment and the food provisioned by the parents with the bacterial assemblage of the nestlings, and the possibility that environmental factors may override host-genetic factors in influencing microbial richness in nestlings.

Micro-environmental factors associated with the anatomical structure of the digestive tract are instrumental in shaping bacterial gut communities (Berg 1996; Stevens and Hume 1998); thus, the composition of the microbial community may be influenced by host lineage, as a consequence of variation in individual phenotypic quality (van der Wielen et al. 2002). In chickens, for example, resistance to *Campylobacter jejuni* and *Salmonella enteritidis* have both been accredited to differences between genetic lineages (Stern et al. 1990; Kaiser and Lamont 2001). Variations evident within blue tit families, where a higher degree of similarity in bacterial community composition was found between siblings than between nestlings and their parents, could be due to host-genetic factors, as the within-pair genetic similarity of adults is lower than the within-brood similarity of nestlings (Mills et al. 1999).

During the winter months, blue tits form roving flocks, but disperse in January and February, when males begin to defend breeding territories (Perrins 1979, 1991). By the time the eggs hatch, breeding pairs have spent a couple of months in the same territory (Perrins 1979), and the strong correlation between the bacterial diversities of breeding pairs suggests that richness is likely to be driven by environmental factors, as microsatellite genotyping in this population found that the relatedness within pairs is low (Leech et al. 2001). However, we found no effect of habitat (*Wood*) on bacterial species richness, suggesting that the similarity among pairs is not due to bacteria derived from their local environment. Both parents provision nestlings with food and remove nestling fecal material from the nest, which provides adult pairs with another shared source of bacterial exposure. Breeding pairs may also share similar bacterial communities as a consequence of bacterial transfer during copulation (Sheldon 1993; Westneat and Rambo 2000; Hupton et al. 2003).

While we have demonstrated that fecal bacterial species richness in blue tits is related to several aspects of the life history of the birds, future research could usefully explore the bacterial community composition, and how different combinations of bacterial OTUs may influence survival and reproductive success.

## Supporting Information

**Table S1.** Field data for all blue tit individuals collected in 2007 and 2008, and binary code representing the bacterial species richness of associated fecal samples.

**Table S2.** Data pertaining to adult blue tit fitness.

**Table S3.** Data pertaining to blue tit chick fitness.

**Table S4.** Fitted values for GLM explaining between-year variation in annual survival of adult blue tits in relation to bacterial species richness (Figure).

**Table S5.** Fitted values for GLM explaining variation in bacterial species richness in adult blue tits in relation to clutch size and clutch initiation date (Figure).

**Table S6.** Data pertaining to individual blue tits sampled in 2007 and re-trapped in 2008.
